# A Novel Pathogenic Variant in *TRAC* Gene Associated with SCID Phenotype: Expanding the Genetic and Clinical Spectrum

**DOI:** 10.1007/s10875-026-02003-3

**Published:** 2026-03-26

**Authors:** Mehmet Ali Karaselek, Mehmet Yavuz Ozbey, Vedat Uygun, Serkan Kuccukturk, Necdet Karabey, Ugur Tokdemir, Gokhan Ozel, Zeynep Dilruba Demircioglu, Serhat Yildirim, Ali Sahin, Tugce Duran, Abdullah Akkus, Selman Yildirim, Sukru Guner, İsmail Reisli, Sevgi Keles

**Affiliations:** 1 Division of Pediatric Immunology and Allergy, Faculty of Medicine, Necmettin Erbakan University, Konya, 42080 Turkey; 2 Pediatric Bone Marrow Transplantation Unit, Department of Pediatric Hematology, Medical Park Hospital, Istinye University Faculty of Medicine, Antalya, 07160 Turkey; 3 Division of Medical Biology, Faculty of Medicine, Karamanoğlu Mehmetbey University, Karaman, 701200 Turkey; 4 Medicine Faculty, Selcuk University, Konya, 42130 Turkey; 5 Division of Medical Genetics, Faculty of Medicine, KTO Karatay University, Konya, 42080 Turkey; 6 Division of Pediatric, Faculty of Medicine, Necmettin Erbakan University, Konya, 42080 Turkey; 7 Division of Medical Genetics, Faculty of Medicine, Necmettin Erbakan University, Konya, 42080 Turkey

**Keywords:** Lymphopenia, Severe Combined Immunodeficiency, T cells, *TRAC*, TCR

## Abstract

**Purpose:**

Pathogenic variants in the T-cell receptor alpha constant (*TRAC*) gene have been primarily associated with combined immunodeficiency (CID). To date, only five patients from three unrelated families harboring the same *TRAC* variant with a CID phenotype, and three patients carrying a distinct variant with severe combined immunodeficiency (SCID), have been described. We report a previously unreported homozygous *TRAC* variant causing a premature stop codon in three siblings with classical SCID phenotype.

**Methods:**

Comprehensive immunological and molecular analyses were performed, including lymphocyte immunophenotyping, proliferation assays, qPCR for T helper (Th) subset–related gene expression, and cytokine secretion profiling. In silico analyses included conservation assessment, structural modeling using ChimeraX, and protein stability prediction via PremPS to evaluate the variant’s structural and functional consequences.

**Results:**

All three siblings exhibited recurrent infections, refractory diarrhea, and elevated liver enzymes, accompanied by profound T-cell lymphopenia with preserved B-cell numbers. Whole-exome sequencing revealed a homozygous *TRAC* variant in the affected siblings and heterozygous carriage in their parents. The variant alters a highly conserved residue, disrupting hydrogen bonding and likely destabilizing the protein structure. Functional assays demonstrated a marked reduction in recent thymic emigrants (RTEs) cell ratio absence of TCRαβ⁺ T cells, skewed Th polarization, and elevated proinflammatory cytokine levelsfindings consistent with a SCID phenotype.

**Conclusion:**

These findings expand the clinical and molecular spectrum of *TRAC*-related immunodeficiency and support its inclusion among genes primarily associated with SCID. The results further emphasize that specific mutation sites within immune-related genes critically influence disease severity and phenotype variability.

**Supplementary Information:**

The online version contains supplementary material available at 10.1007/s10875-026-02003-3.

## Introduction

T cells are a subset of lymphocytes that play a central role in host defense against pathogens and tumors through cell-mediated immunity [1]. The T-cell receptor (TCR) is a multimeric complex on the T-cell surface that is engaged when antigen-presenting cells (APC) display peptide antigens bound to major histocompatibility complex (MHC) molecules, thereby initiating intracellular signaling cascades that culminate in proliferation, differentiation, cytokine secretion, and/or activation-induced cell death [2].

T-cell activation, differentiation, and effector functions are tightly regulated by multiple pathways, beginning with TCR engagement [3]. The antigen-recognition module is the αβ (or γδ) heterodimer, whereas signal transduction is mediated by the CD3 ε, δ, γ chains together with the ζ homodimer. Co-stimulatory receptors further modulate activation thresholds [2]. Upon peptide–MHC ligation, the Src-family kinase Lck associated with CD4 or CD8 phosphorylates immunoreceptor tyrosine-based activation motifs (ITAMs) within the CD3/ζ subunits, recruiting and activating ZAP-70. Downstream phosphorylation of adaptor/scaffold proteins drives calcium mobilization, Ras–MAPK signaling, and cytoskeletal remodeling. Defects affecting the TCR or its downstream signaling components lead to autoimmunity and/or immunodeficiency, ranging in severity from combined immunodeficiency (CID) to severe combined immunodeficiency (SCID) [3–6].

CIDs are inborn errors of immunity characterized by impaired T-cell development, maturation, or function. To date, > 70 genetic defects have been linked to T-cell deficiencies, including > 50 associated with CID [7–10]. On the other hand, SCID are more severe form of T cell deficiency which lead to life-threatening infections and high mortality ratio if not suffered hematopoietic stem cell transplantation in first two years of life [7]. The T cell receptor alpha constant (*TRAC*) gene mutations were classified in CID according to the latest International Union of Immunological Societies (IUIS) classification [9]. A pathogenic *TRAC* variant (c.*+1G > A, p.Trp107Leufs56*) was first reported in 2011 in two unrelated children aged 15 and six months with recurrent infections [11], and the same variant was subsequently identified ten years later in three additional unrelated patients [12]. These five cases fall under the clinical category of CID. A newborn-screen–identified case carrying *TRAC* c.347 C > G (p.Ser116*) exhibited a SCID phenotype with markedly reduced recent thymic emigrants (RTE) [13]. More recently, two further SCID cases with pathogenic *TRAC* variants (c.192_205del; c.361 C > T, p.Arg121*) were reported [6] (Table 2).

Here, we describe three siblings who initially presented with a classical SCID phenotype and were subsequently diagnosed with *TRAC* deficiency due to a previously unreported variant (c.194G > A), resulting in a premature stop codon (p.Trp65*). Two siblings died at three and seven months of age with a SCID-like clinical presentation, whereas the surviving patient is currently well on follow-up after HSCT. These findings expand the *TRAC* variant spectrum and its associated clinical manifestations.

## Patients and Methods

### Study Design and Patients

The study was carried out in 2024 at Necmettin Erbakan University, Department of Pediatric Immunology and Allergy. The flowchart of the study is shown in supplementary Fig. S1. Three patients with *TRAC* gene mutation were evaluated but the detailed immunological studies were conducted with a single patient because the patient’s two siblings died at the age of three and seven months, respectively. Demographic and laboratory data of the patients were evaluated. Five mililiters (ml) blood sample were obtained from patients and healthy controls for flow cytometric and functional analysis.

### Flow Cytometry Analysis and CD25 Activation Assay

Flow cytometric analysis was performed to evaluate naive and memory T cells, NK subsets, RTE, and TCRαβ and TCRγδ ratios. Following monoclonal antibodies were used for surface staining following the appropriate staining protocol; CD4 (PE), CD8 (PC7), CD45RA (PC7), CD45RO (PE), CD31 (FITC), TCRαβ (FITC), TCRγδ (PE), CD3 (PE), CD16 (FITC), CD56 (PerCp) and CD57 (PC5) for immunological analysis and CD3 (PE), CD4 (FITC), CD8 (PerCP), and CD25 (APC) for CD25 activation assay. CD25 activation analysis was performed as previously described [14]. According to our laboratory routine due to profound CD4⁺ T-cell lymphopenia in SCID patients, CD45RA and CD45RO expression analyses were performed within the CD3⁺ T-cell gate before HSCT. Following HSCT, after T-cell compartment, analyses were performed within the CD4⁺ and CD8^+^ T-cell gate. The corresponding gating strategy CD45RA and CD45RO were schematically illustrated in Fig. S2A. The samples were analyzed using a Beckman Coulter flow cytometer (Beckman Coulter, USA), and the data were processed and analyzed with Kaluza analysis software (Beckman Coulter, USA). The samples after HSCT were analyzed on a BD FACSLyric™ Clinical flow cytometer (BD Biosciences, Heidelberg, Germany), and the resulting data were processed with BD FACSuite™ Clinical and BD FACSuite™.

### qPCR Analysis of Helper T (Th) Subsets, NK Receptors and *TRAC*

In qPCR analysis, three primer sets were generated to interrogate regions upstream of the premature termination codon (UP), spanning the mutation site (PTC-spanning), and downstream of the predicted nonsense mutation (Down). Transcription factors specific to Th cell subsets; Th1: *Tbx21*(T-bet), Th2: *GATA3*, Th17: *RORC* (RORγt), Treg: *STAT5*,* FoxP3*), cytokines (Th1: *IFN-γ*; Th2: *IL4*,* IL5*,* IL13*; Th17: *IL17*,* IL21*,* IL22*; Treg: *IL-10*,* TGFβ*), co-stimulatory molecule expressions (*CD28*,* CTLA-4*,* PD1*), NK activation receptors (*NKG2D*,* NKp30*,* NKp44*,* NKp46*), NK inhibition receptors (*NKG2A*,* CD94*,* CD96*,* TIGIT*) proinflammatory cytokines (*IL12*,* IL1β*,* IL6*) and *ZAP70* expressions were evaluated. For this purpose, RNA isolated from peripheral blood mononuclear cells (PBMCs) using the RNA isolation kit [Hibrigen (Turkey)] followed by cDNA synthesis using the cDNAsynthesis kit [Hibrigen (Turkey)] and subsequent qPCR reactions, as previously described, to assess gene expression levels [15], . The primers used in the study were designed using online primer design tools (supplementary Table S1). Primer specificity was verified by UCSC In-Silico PCR and NCBI Primer-BLAST against the human genome (GRCh38), and only primer pairs yielding a single amplicon of the expected size were used. qPCR analyses were conducted in a total volume of 10 µL using the LightCycler 96 (Roche Life Science) qPCR device.

### Amplicon Design Relative to Variant

Based on the pathogenic stop-gain in *TRAC* (c.194G > A; p.Trp65*), we designed three qPCR amplicons positioned relative to the premature termination codon (PTC): UP (upstream of the PTC), PTC (spanning the PTC), and DOWN (downstream of the PTC). UP and DOWN primers were placed away from the PTC to avoid local sequence/context effects, whereas the PTC amplicon centered the variant. Coordinates were referenced to GRCh38 (chr14) and the (ENST00000611116.2) transcript. The primer design strategy and primer sequences are shown in Fig. S3.

### *In-silico* Analysis of Variant

For in silico analyses, we interrogated the human TRAC sequence (UniProt P01848) and the stop-gained variant p.Trp65* (HGVS c.194G > A). Global deleteriousness was retrieved from Combined Annotation Dependent Depletion (CADD) [16], and population frequency was queried in gnomAD (exomes/genomes) with cross-checks in 1000 Genomes/ExAC/dbSNP. Cross-species conservation of the tryptophan at position 65 was assessed by retrieving TRAC protein sequences from 11 species (Ensembl) and performing a multiple-sequence alignment using ClustalΩ in Jalview [17]. Potential nonsense-mediated decay (NMD) was evaluated on the reference transcript (ENST00000611116.2) using the 50–55-nt rule relative to the final exon–exon junction (NMD-positive if > 50–55 nt upstream). Protein-level modeling used the UniProt wild-type (WT) sequence and a truncated sequence representing p.Trp65* (residues 1–64; the stop was excluded from inputs); translation and global alignment confirmed identity across residues 1–64. Membrane features were predicted with SignalP 5.0 (Eukarya), Phobius, and the TOPCONS consensus server (default settings); DeepTMHMM was run for completeness, but interpretation relied on Phobius/TOPCONS. Intrinsic disorder and MoRF propensity were estimated with IUPred2A (long mode; threshold 0.5) and ANCHOR2. Structural figures were prepared in UCSF ChimeraX from the AlphaFold model AF-P01848-F1, coloring residues 1–64 (retained) and 65–141 (lost; 60% transparency) and highlighting the predicted C-terminal transmembrane helix (118–139) as a dark-red tube; for context, the model was superposed onto the α-chain of a representative TCRαβ complex via MatchMaker. Domain annotations were compiled from InterPro/UniProt [18].

### Sanger Sequencing

Genomic DNA was extracted from EDTA-anticoagulated blood using the QIAamp DNA Blood Mini Kit (Qiagen). The *TRAC* target region was PCR-amplified with primers (F: 5′-CTAACCCTGATCCTCTTGTCCC-3′, R:5′-ATAAGGCCGAGACCACCAATC-3′). Amplicons were purified (ExoSAP-IT) and bidirectionally sequenced using BigDye Terminator v3.1 on an ABI 3500 Genetic Analyzer. Chromatograms were aligned to GRCh38, and variants were described according to HGVS nomenclature.

### Hematopoietic Stem Cell Transplantation

The conditioning regimen consisted of treosulfan 10 g m² day¹ for 3 days (days − 7 to − 5), fludarabine 30 mg/m² for 5 days (days − 7 to − 3), and anti-thymocyte globulin (ATG-Fresenius) 5 mg/kg for 3 days (days − 4 to − 2). HSCT delivered 7.0 × 10⁸ mononuclear cells/kg and 8.1 × 10⁶ CD34⁺ cells/kg. Graft-versus-host disease (GVHD) prophylaxis comprised tacrolimus plus methotrexate 10 mg/m² on days + 1, +3, and + 6. Because of potential myelotoxicity that could delay engraftment, ganciclovir was replaced by foscarnet for cytomegalovirus (CMV) management in the early post-transplant period. Neutrophil engraftment was defined as the first of three consecutive days with an absolute neutrophil count (ANC) ≥ 0.5 × 10⁹/L, and platelet engraftment as the first of seven consecutive days with a platelet count ≥ 20 × 10⁹/L in the absence of transfusion [19].

### Statistical Analysis

Changes in gene expression observed in patients and controls were analyzed using the 2^^ΔΔCT^ method [20]. The *β-actin* housekeeping gene was used for normalization. As a result of the analysis, changes in gene expression were expressed as fold-change. The statistical significance of the fold changes was assessed using Student’s t-test with IBM SPSS statistical software (version 21), considering p-values < 0.05 as significant. Visual representation of the results was generated using volcano plots [21] created with the VolcaNoseR online platform.

## Results

### Demographic, Clinical and Laboratory Features

A 3-month-old male (P1), born to consanguineous parents, presented with history of persistent diarrhea lasting a month. The patient was born at 38 weeks with a normal spontaneous vaginal route weighing 3000 gr. Physical examination revealed no positive findings except hepatomegaly (2 cm) and left periauricular skin tag. No significant growth retardation was found. There was no tymus appearance in his Chest X-Ray (supplementary Fig. S4A). Immunological evaluation revealed lymphopenia (min-max: 1800–3630 mm^3^), reduced CD3 + total T cells, CD4 + Th cells, and CD8 + CTLs. CMV DNA was found to be high (1.3 × 10^6^ IU/mL) in blood and stool. Broad-spectrum antibiotherapy and ganciclovir treatment were initiated together with intravenous immunoglobulin replacement therapy (IVIg) with diagnosis of severe combined immunodeficiencies. Tuberculosis prophylaxis could not be started because of elevated transaminases levels in the patient who was vaccinated with BCG. CMV positivity regressed and improved (1 × 10^3^ IU/mL).

In family history, two of the patient’s siblings had died at three (P2) and seven months (P3) of age following prolonged diarrhea, respectively, Further, there was additional deaths under six months of age in the family (4 siblings of the father and 1 sibling of the mother) (Supplementary Fig. S4B). When retrospectively evaluated P2 and P3 clinical and laboratory record, P2 had presented prolonged diarrhea, persistent fever, increased liver enzymes, cytopenia, hepatomegaly, splenomegaly, and an *Enterococcus faecium* infection to another center at three months of age. Immunophenotyping revealed reduced counts of CD3⁺ total T cells, CD4⁺ helper T cells, and CD8⁺ cytotoxic T lymphocytes (Table 1). The IgG level was within the normal range under immunoglobulin replacement therapy (IgRT). P2 had not respond to treatment and had died at follow up with infections. The patient’s other sibling (P3), who died at 7 months of age, had anemia and diarrhea as clinical manifestation. His immunological evaluation revealed lymphopenia, low CD3 + CD4+ and CD3 + CD8+T cells ratio. The P3 developed significantly elevated transaminase levels during follow-up like other siblings and subsequently died with unknown cause when searching for etiology. The immunological profiles of all three siblings are summarized in Table 1.

**Table 1:** Clinical and laboratory characteristics of patients

Parameters	Patient (pre-HSCT)	Patient (post-HSCT)	P2	P3
Age of onset	3 m	11 m	3 m	7 m
Clinical features	Prolonged diarrhea, skin tag on the left ear and hepatomegaly	No clinical findings	Prolonged diarrhea, fever, cytopenia, hepatomegaly, splenomegaly	Prolonged diarrhea, anemia
Infectious Agent	CMV	None	Enterococcus faecium	None
Alanine aminotransferase (u/L)	176 (0–41)	32.3 (0–41)	106 (0–41)	208 (0–41)
Aspartate aminotransferase (u/L)	90 (0–40)	19.6 (0–40)	1004 (0–40)	1508 (0–40)
Blood urea nitrogen (mg/dL)	8 (16.6–48.5)	10.9 (16.6–48.5)	31 (16.6–48.5)	9.9 (16.6–48.5)
Creatinine (mg/dL)	0.38 (0.17–0.42)	0.37 (0.17–0.42)	0.43 (0.17–0.42)	0.11 (0.17–0.42)
White blood cell (count/mm^3^)	3070 (4000–10000)	5710 (4000–10000)	3040 (4000–10000)	2980 (4000–10000)
Neutrophil (count/mm^3^)	820	1840	1570	1090
Platelet (10^3^/uL)	292 (150–400)	336 (150–400)	73 (150–400)	207 (150–400)
Hemoglobin (mg/dL)	8.2 (12.1–17.2)	10 (12.1–17.2)	6.84 (12.1–17.2)	8.5 (12.1–17.2)
Lymphocyte (count/mm^3^)	2000 (2416–9694)	3050 (2965–10471)	1300 (3400–12200)	1730 (3325–9563)
CD3 + T cell (count/mm^3^)	250 (1492–6385)	2592 (1945–7129)	288 (1492–6385)	ne
CD3 + T cell (%)	12.51 (50.4–79.6)	85.05 (50.4–79.6)	22.15 (50.4–79.6)	ne
CD3 + CD4+ Th cell (count/mm^3^)	92 (909–4523)	343.3 (1161–4819)	97 (909–4523)	58 (1190–4481)
CD3 + CD4+ Th cell (%)	4.63 (31.6–57.9)	11.26 (31.6–57.9)	7.5 (31.6–57.9)	3.35 (28.6–59.7)
CD3 + CD8+ CTLs cell (count/mm^3^)	36 (254–2123)	2232 (310–2250)	32 (254–2123)	14 (576–2582)
CD3 + CD8+ Th cell (%)	1.8 (10.7–28.2)	73.20 (10.7–28.2)	2.47 (10.7–28.2)	0.8 (9–31)
CD3-CD16 + CD56+ NK cell (count/mm^3^)	335 (101–1633)	238 (130–1073)	176 (101–1633)	ne
CD3-CD16 + CD56+ NK cell (%)	16.77 (1.8–27.4)	7.8 (1.8–27.4)	13.54 (1.8–27.4)	ne
CD19 B cell (count/mm^3^)	1350 (237–2564)	183 (467–3112)	1278 (237–2564)	1124 (117–2845)
CD19 B cell (%)	67.53 (10.2–36)	6.05 (10.2–36)	98.30 (10.2–36)	65 (5.4–39.6)
CD3 + CD45 RA + T cell %	38.47 (72–93)	7.6 (72–93)	ne	ne
CD3 + CD45 RO + T cell %	5.14 (9–31)	85.12 (9–31)	ne	ne
CD45RA+/CD4+/CD31 + T cell %	3.97 (56.6–90.7)	4.85 (56.5–87.4)	ne	ne
CD3+TCRαβ + T cell %	0 (90.3–99)	87.7 (86.7–97)	ne	ne
CD3+TCRγδ + T cell %	11.9 (0.9-9)	0.44 (2.3–13.5)	ne	ne
IgG (mg/dl)	357 (376–685)	482 (463–1006)	1030*	1390*
IgM (mg/dl)	78 (36–77)	15.3 (46–159)	ne	51
IgA (mg/dl)	49 (9–30)	6.5 (17–69)	ne	11
IgE (IU/dl)	18	< 17	ne	ne
Anti-Hbs (mIU/ml)	Positive	ne	Positive	ne

*HSCT* Hematopoietic stem cell transplantation, *CMV* Cytomegalovirus, *Th* Helper T, *CTLs *Cytotoxic T cells, *NK* Naturel killer cell, *TCR *T cell receptor, *Ig* Immunoglobulin, *m* month, *ne *not evaluated, *: received intravenous immunoglobulin therapy

### Immunological Evaluation

**Fig. 1:**
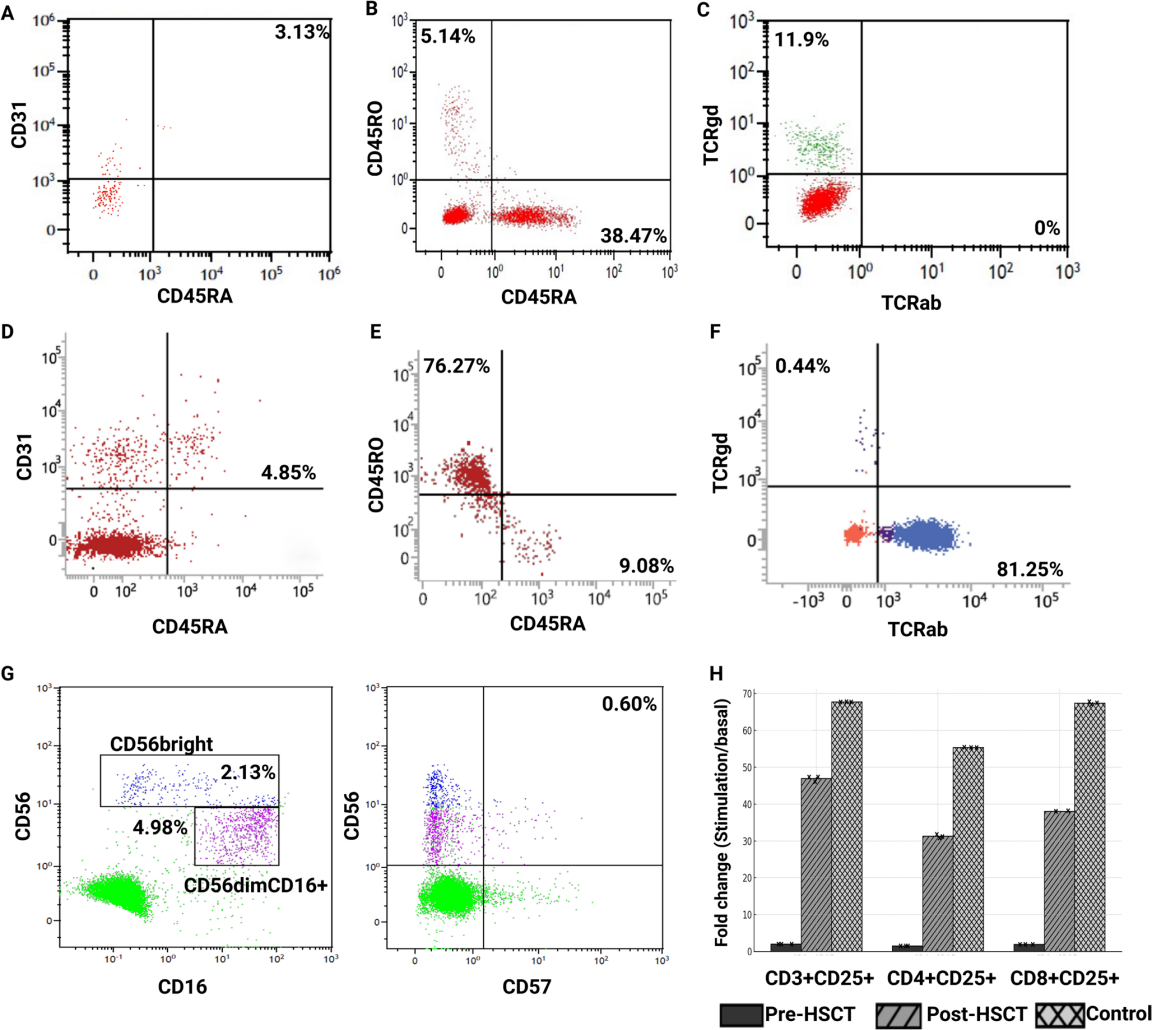
Immunological results of patients and controls. (**A**) Pre-HSCT recent thymic emigrants (Gated on CD4 + cells). (**B**) Pre-HSCT naïve and memory T cells (Gated on CD3 + cells). (**C**) Pre-HSCT T cell receptor (gated on lymphocytes). (**D**) Post-HSCT recent thymic emigrants (Gated on CD4 + cells). (**E**) Post-HSCT naïve and memory T cells (Gated on CD4 + cells). (**F**) Post-HSCT T cell receptor (gated on lymphocytes). (**G**) Pre-HSCT naturel killer subset (Gated on CD3- cells). (**H**) Pre- and post-HSCT CD25 activation assay results. (HSCT: Hematopoietic stem cell transplantation; TCR: T cell receptor)

Flow cytometric analysis of P1 demonstrated a profoundly impaired T-cell compartment consistent with a severe combined immunodeficiency phenotype. The proportion of RTE was markedly reduced at both evaluated time points (3.97% and 3.13%; Fig. 1A), indicating impaired thymic output. Naïve T cells predominated, as indicated by a high proportion of CD3⁺CD45RA⁺ cells (38.47%) accompanied by low memory T-cells (CD3⁺CD45RO⁺, 5.14%) (Fig. 1B). Surface TCR expression analysis revealed complete absence of CD3⁺TCRαβ⁺ cells, while a small population of CD3⁺TCRγδ⁺ cells was detectable (11.9%; Fig. 1C), consistent with defective TCRαβ complex formation. NK cell subsets were preserved, including CD56^bright^, CD56^dim^CD16⁺, and CD56⁺CD57⁺ populations, indicating selective T-cell impairment rather than a global lymphoid defect (Fig. 1G). Functional assessment of T cells showed markedly reduced CD25 upregulation in CD3⁺, CD4⁺, and CD8⁺ T cells compared with controls, supporting impaired T-cell activation (Fig. 1H) Table 2.

**Table 2:** Clinical, immunological and genetic features of the patients having *TRAC* mutation

Parameters [References]	P1 [11]	P2 [11]	P3 [12]	P4 [12]	P5 [12]	P6 [13]	P7 [6]	P8 [6]	This report		
									P1	P2	P3
Age of onset	15mo	6mo	9y	8y	11mo	Newborn	20 days old	< 1 mo old	3mo	3mo	7mo
Clinical features	Recurrent respiratory tract infection, otitis media, candidiasis, diarrhea, and failure to thrive	Recurrent respiratory tract infection, otitis media, candidiasis, diarrhea, and failure to thrive	Recurrent lower respiratory tract infections and multiple cutaneous warts	Recurrent lower respiratory tract infections, multiple cutaneous warts and renal abscess	Recurrent lower respiratory tract infections, left ear discharge, fever and swelling of left eyelid,	nr	Fever, myocarditis, BCG-osis, recurrent CMV infection	Chronic diarrhea, oral thrush, BCG-osis, pneumonia (*Salmonella*), failure to thrive	Prolonged diarrhea, skin tag on the left ear and hepatomegaly	Prolonged diarrhea, fever, cytopenia, hepatomegaly, splenomegaly	Prolonged diarrhea, anemia
Clinical diagnosis	CID	CID	CID	CID	CID	SCID	SCID	SCID	SCID	SCID	SCID
Infectious Agent	Herpes viral infection, varicella, chronic EBV and HHV6 viremia	Varicella	nr	nr	EBV	nr	CMV	*Salmonella*	CMV	*Enterococcus faecium*	ne
Autoimmunity	Yes (low-titer ANA, vitiligo, and alopecia)	Yes (eczema, AIHA, antilymphocyte antibodies, anti-TTG antibodies, low-titer ANA, and pityriasis rubra pilaris)	No	No	No	nr	nr	nr	No	ne	ne
Organomegaly	LAP and HM	LAP and HSM	LAP and HM	LAP	LAP (mature B cell lymphoma)	nr	LAP	No	HM	HSM	No
Mutation	c.*1G > A Nonsense	c.*1G > A Nonsense	c.*1G > A Nonsense	c.*1G > A Nonsense	c.*1G > A Nonsense	c.347 C > G Nonsense	c.192_205del Nonsense	c.361 C > T Nonsense	c.194G > A Nonsense	c.194G > A Nonsense	c.194G > A Nonsense
HSCT	Yes (at 6 y)	Yes (at 6 y)	No	No	No	Yes (at 1 y)	Yes (at 22 mo)	No	Yes (at 22 mo)	No	No
Alive/death	Alive	Alive	Death (due to severe pneumonia)	Death (due to pulmonary complications)	Death (due to pulmonary complications)	Alive	Alive	Death at 3 y (pneumonia)	Alive	Death at 3 mo	Death at 7 mo
Laboratory Findings											
Lymphocyte	4710 Normal	4219 Normal	2960 Normal	1350 Low	1940 Low	1790 Mildly low	1080 Low	2920 Mildly low	2000 Low	1300 Low	1730 Low
CD3 + T cell (count/mm^3^) / (%)	2356 (50%) Normal	886 (21%) Low	nr (45.1%) Low	nr (43%) Low	nr (2.4%) Low	nr Low	1314 (nr) Low	nr (15%) Low	250 (12.51%) Low	288 (22.5%) Low	ne
CD3 + CD4+ Th cell (count/mm^3^) / (%)	1293 (27%) Normal	364 (8%) Low	nr (32.6%) Normal	nr (24.4%) Low	nr (6.7%) Low	113 (nr) Low	nr Low	nr (7%) Low	92 (4.63%) Low	97 (7.5) Low	58 (3.35%) Low
CD3 + CD8+ CTLs cell (count/mm^3^) / (%)	911 (19%) Normal	291 (7%) Low	nr (8.2%) Low	nr (20%) Normal	nr (20.36%) Normal	76 (nr) Low	nr Low	nr (8%) Low	36 (1.8%) Low	32 (2.47) Low	14 (0.8%) Low
CD3-CD16 + CD56+ NK cell (count/mm^3^) / (%)	851 (18%) Normal	859 (20%) Normal	nr (7.2%) Normal	nr	nr (3.55%) Normal	710 (nr) Normal	350 (nr) Normal	nr (8%) Normal	335 (16.77%) Normal	176 (13.54%) Normal	ne
CD3+TCRγδ+ (%)	80^˄^	45^˄^	92.4^˄^	93.3^˄^	98.2^˄^	nr	95^˄^	64^˄^	0	ne	ne
CD3+TCRαβ+ (%)	ne	ne	2.7^˅^	1.8^˅^	1.3^˅^	Absent	Absent	Absent	11.9 ^˅^	ne	ne
CD45RA+CD27+	Low	Low	nr	nr	nr	High	nr	nr	Low	ne	ne
CD19 B cell (count/mm^3^) / (%)	1082 (23%) Normal	2366 (55%) Normal	3.9% Low	8.1% Low	36.87% Normal	638 Normal	1250 Normal	74% Normal	1350 (67.53%) Normal	1278 (98.3%) Normal	1124 (65%) Normal
Memory B cell	Normal	Normal	nr	nr	nr	nt	nr	nr	ne	ne	ne
T cell proliferation assays (with PHA and anti-CD3)	Less than control	Less than control	nr	nr	nr	Less than control	nr	nr	Less than control	ne	ne
IgG (mg/dl)	1572 Normal	712 Normal	2109 Normal	1607 Normal	1094 Normal	nr	ne	220 Low	357 Low	1030*	1390*
IgM (mg/dl)	169 Normal	126 Normal	90 Normal	136 Normal	ne Normal	nr	ne	45 Normal	78 Normal	ne	51
IgA (mg/dl)	123 Normal	69 Normal	456 High	568 High	145 High	nr	ne	Undetectable	49 High	ne	11
IgE (IU/ml)	9187 High	17 Normal	6000 High	4392 High	1844 High	nr	nr	nr	18 Normal	ne	ne

*AIHA* Autoimmune hemolytic anemia, *EBV* Epstein–Barr virus, *HHV6* Human herpesvirus 6, *BCG* Bacillus Calmette–Guérin *ANA* antinuclear antibodies, *TTG* Tissue transglutaminase antibodies, *Th* Helper T cell, *CTLs* Cytotoxic T cell, *NK* Natural killer cell, *TCR* T cell receptor, *PHA* Phytohemagglutinin, *LAP* Lymphadenopathy, *HM* Hepatomegaly, *HSM* Hepatosplenomegaly, *Ig* Immunoglobulin, *ne* Not evaluated, *nr* Not reported, *˄* Above normal value, *˅* Below normal value, *mo* month, *y* years

**Fig. 2:**
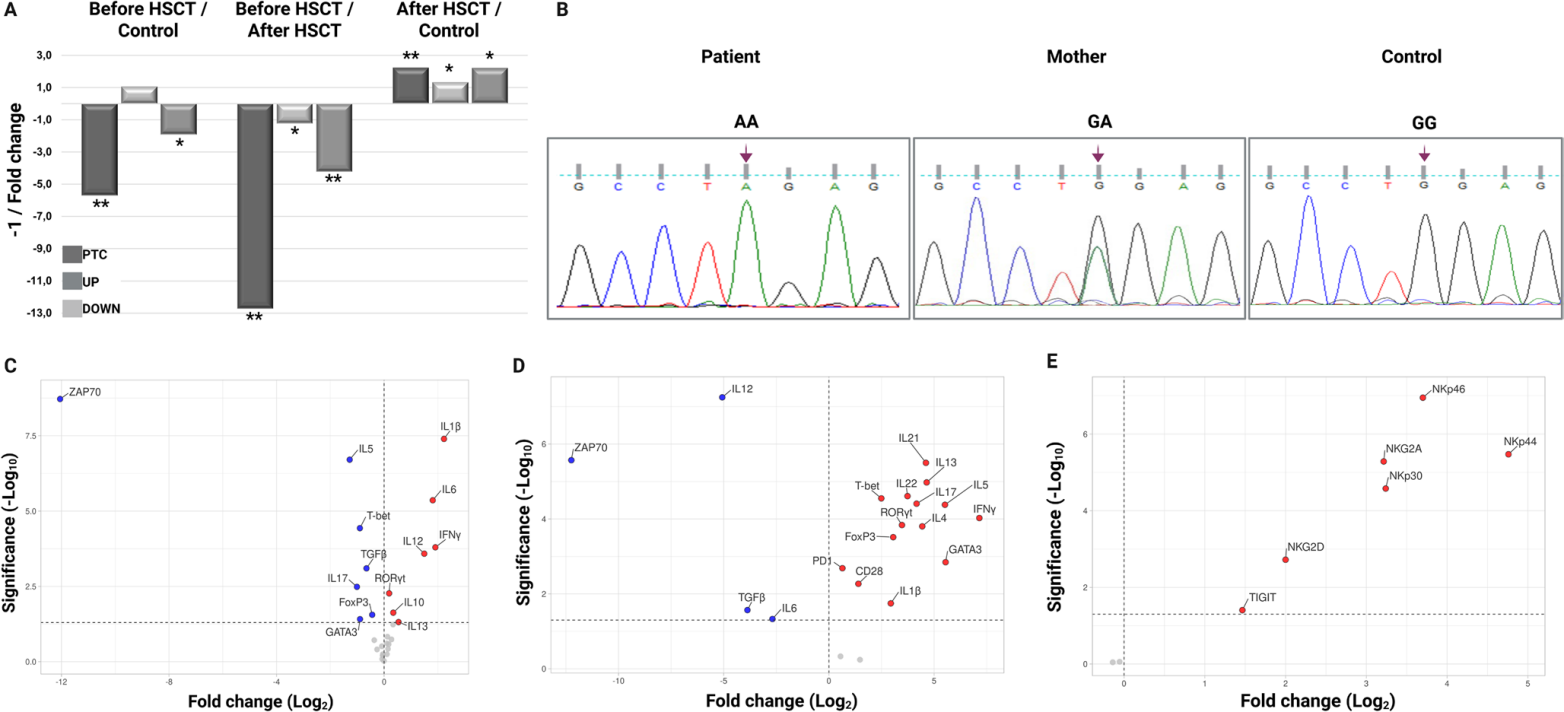
qPCR analysis results of *TRAC* gene variant. (**A**) Comparative *TRAC* expression using amplicons positioned relative to the PTC. (**B**) Sanger sequencing chromatograms of the *TRAC* gene showing the c.194G > A (p.Trp65Ter) variant (**C**) Comparison of gene expression changes of Th subsets and proinflammatory cytokines in the pre-HSCT period with controls. (**D**) Comparison of gene expression changes of Th subsets and proinflammatory cytokines in pre- and post-HSCT period. (**E**). Comparison of gene expression changes of NK activation and inhibition receptors in pre- and post-HSCT period. Red dots: increased significant expression; Blue dots: decreased significant expression; Grey dots: insignificant change expression/unchanged. For volcano plots, the right side of the “0” point on the fold-change (Log2) axis represents increased expression and the left side represents decreased expression. The value of “1.3"on the significance (-Log10) axis indicates *p* < 0.05 at the top and *p* > 0.05 at the bottom. (HSCT: Hematopoietic stem cell transplantation; PTC: Premature termination codon; UP: upstream PTC; Down: Downstream of PTC; **: *p* < 0.001; *p < 0.01)

Quantitative PCR analysis using three *TRAC* amplicons positioned relative to the premature termination codon (UP, PTC, and Down) demonstrated a selective and profound reduction of transcripts spanning the PTC region before HSCT, with comparatively milder changes upstream and downstream of the variant. Following HSCT, expression of all three amplicons was restored and exceeded control levels, indicating reconstitution of TRAC transcript expression. The disproportionate reduction of the PTC-spanning amplicon prior to HSCT is consistent with selective depletion of mutant transcripts (Fig. 2A).

Analysis of transcription factors and cytokines associated with Th cell subsets revealed broad dysregulation of adaptive immune responses. Key transcription factors and cytokines related to Th1, Th2, Th17, and regulatory T-cell lineages were significantly downregulated, whereas *IFN-γ* and *IL-10* were upregulated, reflecting an imbalanced cytokine milieu. Proinflammatory cytokines, including *IL-6*,* IL-12*, and *IL-1β*, were markedly increased. In contrast, NK receptor gene expression remained comparable to controls, further supporting selective T-cell–restricted immune dysfunction (Fig. 2C).

### Hematopoietic Stem Cell Transplantation Outcomes

The patient underwent HSCT with grafts obtained from a 9/10 HLA-matched unrelated donor. The clinical and laboratory findings after HSCT are summarized in Table 1. Neutrophil engraftment was documented on day + 17 and platelet engraftment on day + 8. CMV viremia cleared by day + 27, allowing cessation of antiviral therapy. Hepatic graft-versus-host disease emerged on day + 75, prompting the introduction of mycophenolate mofetil in addition to tacrolimus. At the 6-month evaluation the patient exhibited sustained full donor chimerism. HSCT completely resolved the patient’s neutropenia and lymphopenia and markedly improved all major lymphocyte subsets (Table 1). As the assessment was performed in the early post-HSCT period, the proportion of RTE did not change appreciably (Fig. 1D). Following HSCT, both CD4⁺ and CD8⁺ T-cell compartments exhibited a shift toward a CD45RO⁺ memory phenotype, with a marked reduction in CD45RA⁺ naïve cells (CD4⁺CD45RA⁺ 9.08% vs. CD4⁺CD45RO⁺ 76.27%; Fig. 1E). TCRγδ⁺ T-cell counts normalized (Fig. 1F). Finally, surface CD25 expression increased significantly after HSCT compared with pre-HSCT levels (*p* < 0.001) (Fig. 1H). Following HSCT, ZAP70 expression in Th cells significantly increased (2.42-fold, *p* = 0.001). Transcription factors associated with Th1, Th2, and Th17 subsets also showed a significant upregulation, while pro-inflammatory cytokine expression was markedly decreased (Fig. 2D). In addition, the expression levels of NK cell receptors were significantly elevated in the post-HSCT period (Fig. 2E). Through follow-up, no CMV reactivation or other viral infections were detected despite the absence of scheduled intravenous immunoglobulin replacement.

### Genetic Findings

Whole-exome sequencing had performed in two affected siblings and their parents, revealing a homozygous c.194G > A variant in the *TRAC* gene in both siblings, while the parents were identified as heterozygous carriers of the same variant. The variant was initially classified as a variant of uncertain significance. Clinical exome sequencing of the index case confirmed the same homozygous *TRAC* variant. Sanger sequencing confirmed the homozygous c.194G > A substitution in the patient and heterozygous carrier status in the mother, while the control sample showed a wild-type allele (Fig. 2B).

### *In-Silico* Analysis Results of *TRAC* Gene Variant

**Fig. 3:**
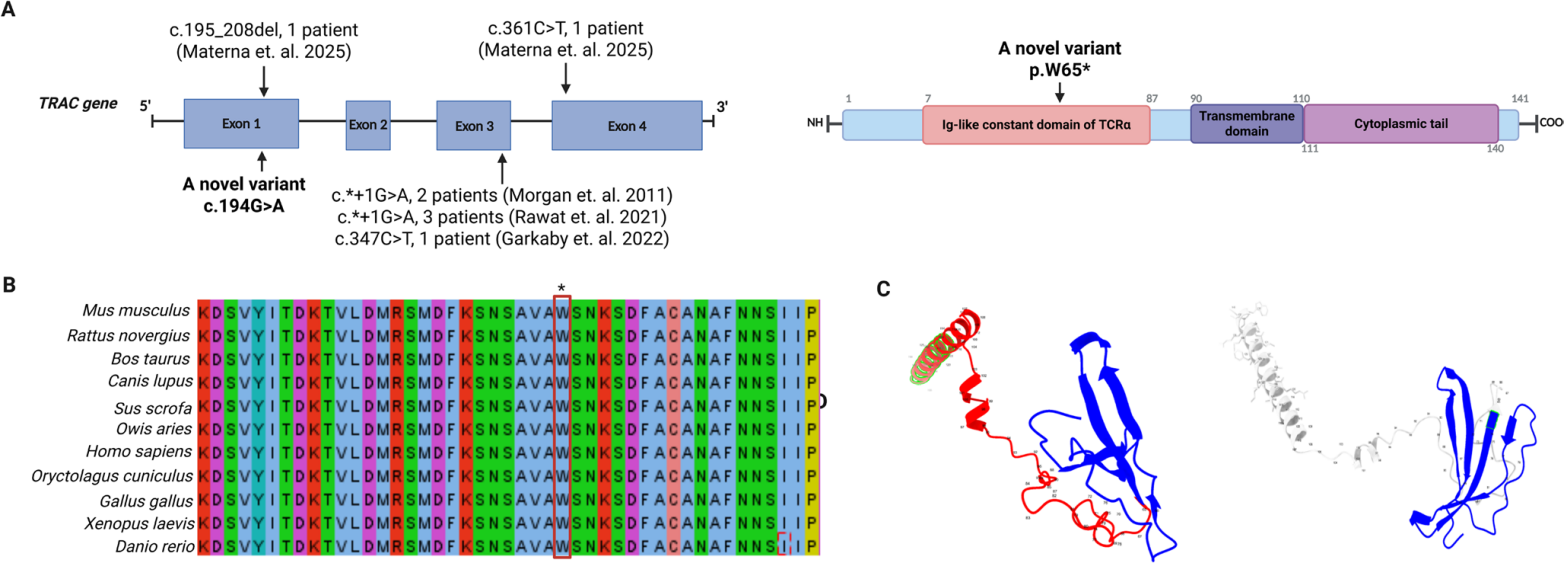
In-silico analysis results of *TRAC* gene variant. (**A** Schematic representation of the *TRAC* gene and protein, highlighting the localization of the c.194G > **A** (p.Ser65Asn) variant at both the gene and protein levels and other *TRAC* gene variants. (**B**) Conservation analysis of the 65 amino acids identified in the *TRAC* gene. (**C**) Intramolecular hydrogen bonding in wildtype and mutant TRAC protein. (**D**) Structural modeling of the TRAC. Left, WT model showing residues 1–64 in blue, residues 65–141 in red (semi-transparent), and the predicted C-terminal transmembrane helix (residues 118–139) as a dark red tube. Right. Mutant p.Trp65* truncation model showing only residues 1–64 (blue), with complete loss of the connecting peptide, TM helix, and cytoplasmic tail. This structural disruption is consistent with nonsense-mediated decay and/or loss of membrane anchoring

The identified nucleotide-level variant in the *TRAC* gene, c.194G > A, was in exon 1 and results in a premature stop codon (p.Trp65*) within the constant region of TCRα (Fig. 3A). The stop-gained variant *TRAC* p.Trp65* (c.194G > A) constitutes a predicted loss-of-function allele, with a CADD v1.7 Phred of 39 (top ~ 0.01% genome-wide). In population datasets, the variant is ultra-rare in gnomAD (AC = 2; AF = 2.84 × 10⁻⁵; cross-checked against 1000 Genomes/ExAC/dbSNP). A multi-species alignment showed that the tryptophan at position 65 is highly conserved, supporting functional importance of the affected residue (Fig. 3B). NMD analysis on the reference transcript (ENST00000611116.2) placed the premature termination codon well > 50–55 nt upstream of the final exon–exon junction, predicting NMD-positive decay of the mutant transcript.

At the protein level, translation and global alignment confirmed a truncated product comprising residues 1–64. Phobius and TOPCONS predicted a single C-terminal TM helix in WT (≈ 118–139) and no TM in the truncated sequence, consistent with loss of membrane anchoring; both WT and truncated sequences lacked a signal peptide (as expected for the TRAC constant region) (Fig. S4A-B). IUPred2A/ANCHOR2 profiles indicated that WT Cα is largely ordered with very low disorder over the TM region, whereas the 64-aa fragment lacks the TM signature and shows only modest N-terminal flexibility without strong MoRF signals (Fig. S5C). InterPro/UniProt annotations and ChimeraX/AlphaFold visualization demonstrated that truncation removes residues 65–141, including the connecting peptide, the entire TM helix, and the short cytoplasmic tail, thereby preventing TCRαβ/CD3 complex assembly at the cell surface. Collectively, these findings support a pathogenic, loss-of-function effect via NMD and/or absence of a stable, membrane-anchored TRAC protein.

Structural modeling revealed that the p.Trp65* variant produces a truncated TRAC protein lacking residues 65–141, including the C-terminal connecting peptide, the transmembrane helix (118–139), and the short cytoplasmic tail (Fig. 3D). When superposed onto the α-chain of the TCRαβ complex (PDB 6JXR), the truncated model retained only the N-terminal 64 residues (blue), while the missing region (red) encompassed the entire membrane-anchoring segment (magenta), consistent with loss of surface expression and failure to assemble a stable TCRαβ/CD3 complex.

## Discussion

Hypomorphic mutations in several of the genes or their alleles that cause SCID may result in broad clinical spectrum, including Omenn syndrome (OS), “leaky” SCID or CIDs. Such phenotypic variability has been well documented in genes involved in V(D)J recombination, particularly *DCLRE1C* and *RAG1/2*, where allelic diversity frequently leads to milder or atypical presentations at different ages [14, 22]. While pathogenic variants in the *TRAC* gene have been reported far less frequently. To date pathogenic *TRAC* variant associated with CID was identified in five patients from two unrelated families [11], whereas only three patients waith a classical SCID phenotype have been reported [11–13]. In present study, we describe three siblings presenting with a severe clinical and immunological phenotype characterized by refractory diarrhea, increased liver enzymes, cytopenias profound T-cell lymphopenia, and absent TCRαβ expression, consistent with classical SCID. Together with recently reported SCID cases carrying pathogenic *TRAC* variants, our findings support the inclusion of *TRAC* among the genetic defects capable of causing a classical SCID phenotype, like *DCLRE1C* and *RAG*-related disorders.

Clinical features reported in the patients with *TRAC*-related CID include skin manifestations, autoimmunity, eosinophilia, vitiligo, warts, and lymphadenopathy, resembling other T-cell immunodeficiencies [14, 22, 23]. In contrast, no major or defining clinical feature has been established to date for the *TRAC*-related SCID. In our patients, the predominant manifestations were recurrent infections-particularly refractory diarrhea- and organomegaly. Immunological evalution revealed lymphopenia, reduced T-cell counts, decreased proportions of RTE, complete absence of CD3+TCRαβ *+* cells, and a relative increase CD3+TCRγδ cell. These findings suggest that assessment of TCRαβ and TCRγδ + expressions by flow cytometry may be informative in patients with clinical and laboratory findings suggestive of T-B+ SCID. Elevated liver enzyme levels observed in our patients could not be conclusively attributed to either infections complications or the underlying disease.

The *TRAC* variant is located within the constant region of TCRα, which plays critical role in maintaining TCR stability and function [11, 12]. The identified *TRAC* variant is predicted to result in a premature termination codon, consistent with a loss-of-function mechanism. For such variants, the primary molecular consequence is expected to be nonsense-mediated mRNA decay, leading to selective depletion of mutant *TRAC* transcripts rather than production of a structurally altered protein. In line with this mechanism, our quantitative PCR analysis demonstrated a marked reduction of transcripts spanning the premature termination codon prior to HSCT, while regions upstream and downstream were less affected. Restoration of *TRAC* transcript levels following HSCT further supports loss of functional *TRAC* expression as the principal pathogenic mechanism underlying the observed immunological phenotype. The observed family segregation pattern, combined with profound T-cell lymphopenia, reduced RTE, and absence of CD3+TCRαβ + cells, supports the pathogenicity of the identified variant. Although *TRAC*-associated immunodeficiency is currently classified by the IUIS as generally less profound than SCID [9], our patients harboring a previously unreported nucleotide-level *TRAC* variant exhibited clinical and laboratory features characteristic of typical SCID.

Functional analyses further demonstrated impaired adaptive immune responses, including *TRAC*, *ZAP70*,* CD28* and Treg cell-associated markers, while NK cell receptor expression remained comparable to control. Transcription factors and cytokine expressions associated with Th1, Th2, Th17, and Treg lineages were decreased, whereas *IFN-γ*,* IL10* and proinflammatory cytokine such as *IL-1*,* IL-1β*,* IL-12* was elevated, likely reflecting innate immune activation [24–26].

HSCT has been performed in a limited number of patients with *TRAC* mutations, including two with CID and two with SCID phenotypes [6, 13]. Although decision-making regarding HSCT is challenging in rare disorders, available evidence-including outcomes from our patients and previously reported cases suggests that HSCT is well tolerated and should be considered promptly once a classical SCID phenotype is recognized.

## Conclusions

In conclusion, consistent with our previous observations in Artemis deficiency [14], the *TRAC* variant identified our patients associated with a classical SCID phenotype. These findings support the consideration of *TRAC* as a first-tier gene in the genetic evaluation of typical SCID. Incorporation of TCRαβ/TCRγδ analysis into diagnostic flow-cytometry panels may facilitate early recognition of the characteristic immunophenotype and expedite molecular diagnosis. Continued identification pathogenic *TRAC* variants, together with detailed clinical annotation, will further refine the phenotypic spectrum of *TRAC* deficiency.

## Supplementary Information

Below is the link to the electronic supplementary material.

Supplementary Material 1 (PDF 759 KB)

## Data Availability

No datasets were generated or analysed during the current study.

## References

[1] Gouaillard C, Huchenq-Champagne A, Arnaud J, Chen Cl CL, Rubin B. Evolution of T cell receptor (TCR) alpha beta heterodimer assembly with the CD3 complex. Eur J Immunol. 2001;31:3798–805. 11745401 10.1002/1521-4141(200112)31:12<3798::aid-immu3798>3.0.co;2-z

[2] Mariuzza RA, Agnihotri P, Orban J. The structural basis of T-cell receptor (TCR) activation: An enduring enigma. J Biol Chem. 2020;295:914–25. 31848223 10.1074/jbc.REV119.009411 PMC6983839

[3] Krangel MS. Mechanics of T cell receptor gene rearrangement. Curr Opin Immunol. 2009;21:133–9. 19362456 10.1016/j.coi.2009.03.009 PMC2676214

[4] Kent A, Longino NV, Christians A, Davila E. Naturally Occurring Genetic Alterations in Proximal TCR Signaling and Implications for Cancer Immunotherapy. Front Immunol. 2021;12:658611. 34012443 10.3389/fimmu.2021.658611 PMC8126620

[5] Ashouri JF, Lo WL, Nguyen TTT, Shen L, Weiss A. ZAP70, too little, too much can lead to autoimmunity. Immunol Rev. 2022;307:145–60. 34923645 10.1111/imr.13058 PMC8986586

[6] Materna M, Seyedpour S, Le Voyer T, Parvaneh N, Yazdanpanah N, Hamidieh AA et al. Two different forms of inherited human TCRα chain deficiency. J Hum Immun. 2025;1(2):e20250014. 10.70962/jhi.20250014 10.70962/jhi.20250014 PMC12526356 41103553

[7] Bousfiha A, Moundir A, Tangye SG, Picard C, Jeddane L, Al-Herz W, et al. The 2022 Update of IUIS Phenotypical Classification for Human Inborn Errors of Immunity. J Clin Immunol. 2022;42:1508–20. 36198931 10.1007/s10875-022-01352-z

[8] Tangye SG, Al-Herz W, Bousfiha A, Cunningham-Rundles C, Franco JL, Holland SM, et al. Human Inborn Errors of Immunity: 2022 Update on the Classification from the International Union of Immunological Societies Expert Committee. J Clin Immunol. 2022;42:1473–507. 35748970 10.1007/s10875-022-01289-3 PMC9244088

[9] Aranda CS, Gouveia-Pereira MP, da Silva CJM, Rizzo M, Ishizuka E, de Oliveira EB, et al. Severe combined immunodeficiency diagnosis and genetic defects. Immunol Rev. 2024;322:138–47. 38287514 10.1111/imr.13310

[10] Poli MC, Aksentijevich I, Bousfiha AA, Cunningham-Rundles C, Hambleton S, Klein C et al. Human inborn errors of immunity: 2024 update on the classification from the International Union of Immunological Societies Expert Committee. J Hum Immun. 2025;1(1):e20250003. 10.70962/jhi.20250003 10.70962/jhi.20250003 PMC12829761 41608114

[11] Morgan NV, Goddard S, Cardno TS, McDonald D, Rahman F, Barge D, et al. Mutation in the TCRα subunit constant gene (TRAC) leads to a human immunodeficiency disorder characterized by a lack of TCRαβ + T cells. J Clin Investig. 2011;121:695–702. 21206088 10.1172/JCI41931 PMC3026716

[12] Rawat A, Singh A, Dobbs K, Pala F, Delmonte OM, Vignesh P, et al. Skewed TCR Alpha, but not Beta, Gene Rearrangements and Lymphoma Associated with a Pathogenic TRAC Variant. J Clin Immunol. 2021;41:1395–9. 33909184 10.1007/s10875-021-01047-x PMC8316983

[13] Garkaby J, Fuentes L, Willett Pachul J, Watts-Dickens A, Fraser M. A Novel mutation in TRAC in a patient with abnormal newborn screening for severe combined immunodeficiency. LymphoSign J. 2022;9(1):5–10. 10.14785/lymphosign-2022-0001

[14] Hazar E, Karaselek MA, Kapakli H, Dogar O, Kuccukturk S, Uygun V, et al. Variable clinical presentation of hypomorphic DCLRE1C deficiency from childhood to adulthood. Pediatr allergy immunology: official publication Eur Soc Pediatr Allergy Immunol. 2024;35:e14260. 10.1111/pai.14260 39425552

[15] Karaselek MA, Duran T, Kuccukturk S, Hazar E, Dogar O, Kıykım A, et al. Molecular investigations on T cell subsets in patients affected by hypomorphic DCLRE1C mutation. Expert Rev Clin Immunol. 2025;21:393–9. 38706114 10.1080/1744666X.2024.2352479

[16] Kircher M, Witten DM, Jain P, O’Roak BJ, Cooper GM, Shendure J. A general framework for estimating the relative pathogenicity of human genetic variants. Nat Genet. 2014;46:310–5. 24487276 10.1038/ng.2892 PMC3992975

[17] Clamp M, Cuff J, Searle SM, Barton GJ. The Jalview Java alignment editor. Bioinf (Oxford England). 2004;20:426–7. 10.1093/bioinformatics/btg430 14960472

[18] Blum M, Andreeva A, Florentino LC, Chuguransky SR, Grego T, Hobbs E, et al. InterPro: the protein sequence classification resource in 2025. Nucleic Acids Res. 2025;53:D444–56. 39565202 10.1093/nar/gkae1082 PMC11701551

[19] Sureda A, Carpenter PA, Bacigalupo A, Bhatt VR, de la Fuente J, Ho A, et al. Correction: Harmonizing definitions for hematopoietic recovery, graft rejection, graft failure, poor graft function, and donor chimerism in allogeneic hematopoietic cell transplantation: a report on behalf of the EBMT, ASTCT, CIBMTR, and APBMT. Bone Marrow Transplant. 2024;59:1335. 38907026 10.1038/s41409-024-02336-w PMC11368801

[20] Livak KJ, Schmittgen TD. Analysis of relative gene expression data using real-time quantitative PCR and the 2(-Delta Delta C(T)) Method. Methods (San Diego Calif). 2001;25:402–8. 11846609 10.1006/meth.2001.1262

[21] Goedhart J, Luijsterburg MS. VolcaNoseR is a web app for creating, exploring, labeling and sharing volcano plots. Sci Rep. 2020;10:20560. 33239692 10.1038/s41598-020-76603-3 PMC7689420

[22] Dobbs K, Tabellini G, Calzoni E, Patrizi O, Martinez P, Giliani SC, et al. Natural Killer Cells from Patients with Recombinase-Activating Gene and Non-Homologous End Joining Gene Defects Comprise a Higher Frequency of CD56(bright) NKG2A(+++) Cells, and Yet Display Increased Degranulation and Higher Perforin Content. Front Immunol. 2017;8:798. 28769923 10.3389/fimmu.2017.00798 PMC5511964

[23] Keles S, Charbonnier LM, Kabaleeswaran V, Reisli I, Genel F, Gulez N, et al. Dedicator of cytokinesis 8 regulates signal transducer and activator of transcription 3 activation and promotes T(H)17 cell differentiation. J Allergy Clin Immunol. 2016;138:1384–94.e2. 27350570 10.1016/j.jaci.2016.04.023 PMC5099100

[24] Liroff K, Fleury C, Dumitrescu C, Kassaye S. Delayed Onset of COVID-19 in an Immunosuppressed Patient. Infect Dis Clin Pract (Baltimore Md). 2021;29:e448–50. 10.1097/IPC.0000000000001027 PMC8594382 34803351

[25] Yang W, Chen X, Hu H. CD4(+) T-Cell Differentiation In Vitro. Methods in molecular biology. (Clifton NJ). 2020;2111:91–9. 10.1007/978-1-0716-0266-9_8 31933201

[26] Cifaldi C, Cotugno N, Di Cesare S, Giliani S, Di Matteo G, Amodio D, et al. Partial T cell defects and expanded CD56(bright) NK cells in an SCID patient carrying hypomorphic mutation in the IL2RG gene. J Leukoc Biol. 2020;108:739–48. 32392633 10.1002/JLB.5MA0220-239R

