# Supplementary material for: A Novel Pathogenic Variant in TRAC Gene Associated with SCID Phenotype: Expanding the Genetic and Clinical Spectrum
Source: J Clin Immunol. 2026 Mar 26;46(1):45. doi: 10.1007/s10875-026-02003-3 (PMC13139271; doi:10.1007/s10875-026-02003-3)
Supplement: Supplementary file 1 — Supplementary Material 1 (PDF 759 KB) [file 10875_2026_2003_MOESM1_ESM.docx]

Table S1. Primer sequences of the helper T cell transcription factor, cytokine, costimulatory molecule, NK cell activation and inhibition genes used in the study

| **Genes** | **Primers (5’…….3’)** | **Genes** | **Primers (5’…….3’)** |
| --- | --- | --- | --- |
| *IFN-γ* | F:GGCAAGGCTATGTGATTACAAGG  R:CATCAAGTGAAATAAACACACAACCC | *IL-6* | F:CCTGAACCTTCCAAAGATGGC  R:TTCACCAGGCAAGTCTCCTCA |
| *T-bet* | F:GTCCAACAATGTGACCCAGAT  R:ACCTCAACGATATGCAGCCG | *IL-12* | F:CCTTGCACTTCTGAAGAGATTGA  R:ACAGGGCCATCATAAAAGAGGT |
| *GATA3* | F:GCCCCTCATTAAGCCCAAG  R:TTGTGGTGGTCTGACAGTTCG | *CD28* | F: CTATTTCCCGGACCTTCTAAGCC  R: GCGGGGAGTCATGTTCATGTA |
| *IL-4* | F:CGGCAACTTTGTCCACGGA  R:TCTGTTACGGTCAACTCGGTG | *PD-1* | F: CAGCCGGCCAGTTCCAAAC  R: CTCCTATTGTCCCTCGTGCG |
| *IL-5* | F:AAGAGACCTTGGCACTGCTTTC  R:GGAACAGGAATCCTCAGAGTCTCA | *CTLA-4* | F: CCCACCGCCATACTACCTGG  R: TGGGCACGGTTCTGGATCAAT |
| *IL-13* | F:GAGGATGCTGAGCGGATTCTG  R:CACCTCGATTTTGGTGTCTCG | *NKG2D* | F: ATTCTAGATCAGGAACTGAGGACA  R: TGCACAAAGGATTCCTGCTCA |
| *RORγt* | F:CTGCTGAGAAGGACAGGGAG  R:AGTTCTGCTGACGGGTGC | *NKp30* | F: GGTGGTGGAGAAAGAACATCCT R: TTGGGGGAATCCGGAGAGAGT |
| *IL-17* | F:TCCCACGAAATCCAGGATGC  R:GGATGTTCAGGTTGACCATCAC | *NKp44* | F: CCTCTCCCCTTCCTGTCCCTCT  R: TGTGCCGATTCCTTAAAACCCACC |
| *IL-21* | F:TAGAGACAAACTGTGAGTGGTCA  R:GGGCATGTTAGTCTGTGTTTCTG | *NKp46* | F: AGCACTAGGCCGGCAGAATC  R: TGACTCAGACACAGCCCGAC |
| *IL-22* | F:GCTTGACAAGTCCAACTTCCA  R:GCTCACTCATACTGACTCCGT | *NKG2A* | F: GCTCATTGTTGGGATCCTGGG  R: GGCCACAATGACGTGAGGGA |
| *FoxP3* | F:GTGGCCCGGATGTGAGAAG  R:GGAGCCCTTGTCGGATGATG | *CD94* | F: GCTCAGCTTCAACAATTCAACGC  R: CACAGAGTGGTCTTAAACACTGCC |
| *STAT5* | F:ACGGGGTGATGGAGGTGTTG  R:TCAGGTTCCACAGGTTGCGT | *CD96* | F: ATGCATGGTCGGTGGAGGAT  R: TGGCAAGAGAACTTCCGCCC |
| *IL-10* | F:TCAAGGCGCATGTGAACTCC  R:GATGTCAAACTCACTCATGGCT | *TIGIT* | F: CTCCCCTCGCCTCAGGAATG  R: TCCTGCTGCTCCCAGTTGAC |
| *TGF-β* | F:CCCAGCATCTGCAAAGCTC  R:GTCAATGTACAGCTGCCGCA | *ZAP70* | F: AAGTTCCTGGTCGGCAAGAG  R:GGTCACGGTGCACAAAGTTC |
| *UP_TRAC* | F:GTCTGTCTGCCTATTCACCGA  R:TGCTCTTGAAGTCCATAGACCT | *PTC_TRAC* | F:TGGACTTCAAGAGCAACAGTG  R:GGCTGGGGAAGAAGGTGTC |
| *Down_TRAC* | F:CCAGCCCAGAAAGTTCCTGT  R:TAAACCCGGCCACTTTCAGG |  |  |


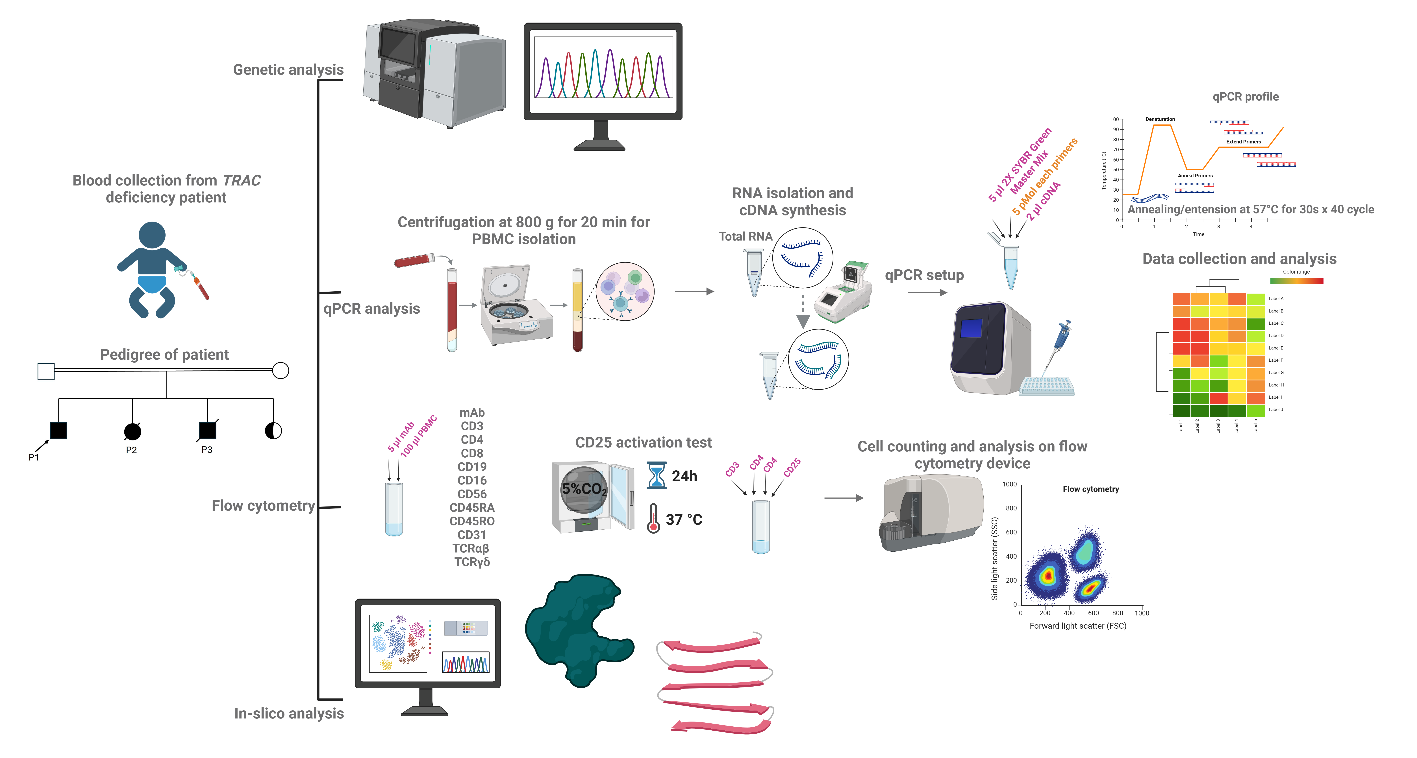


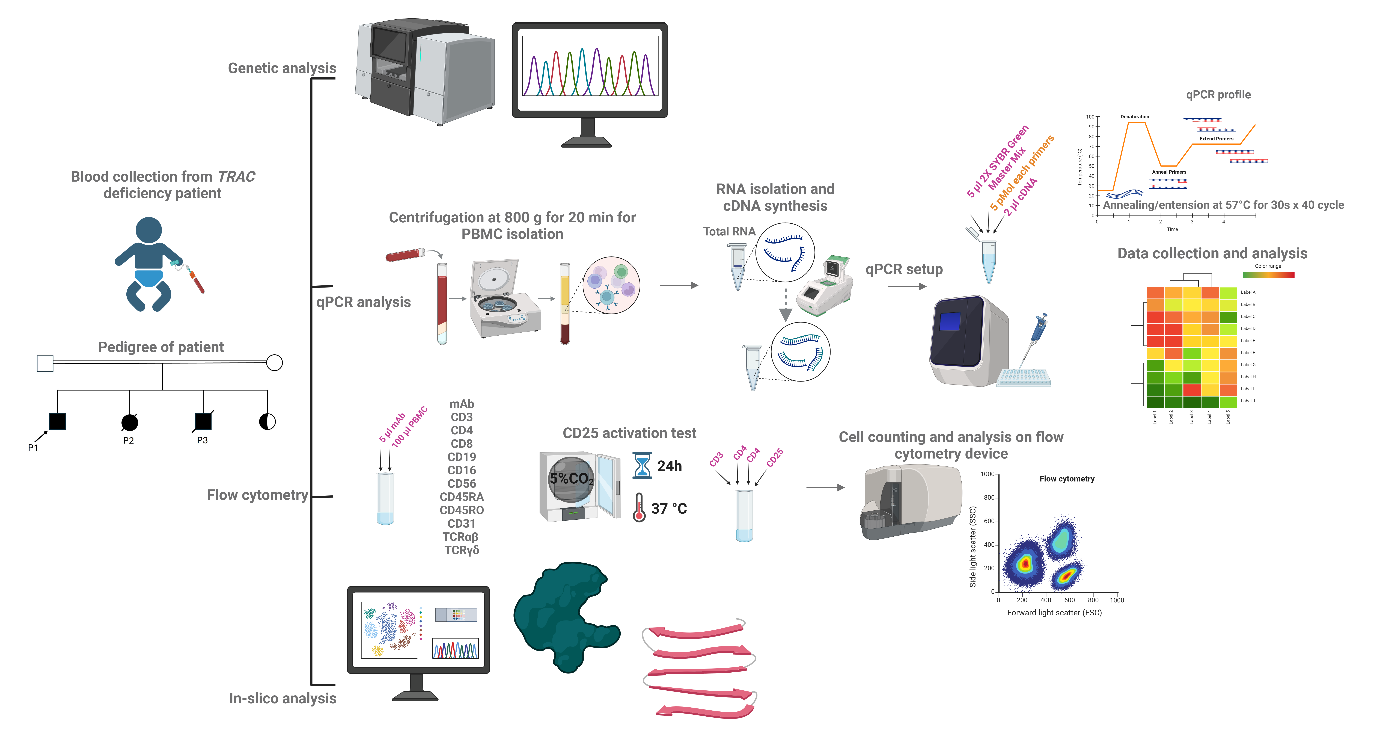


Figure S1. The flowchart of study


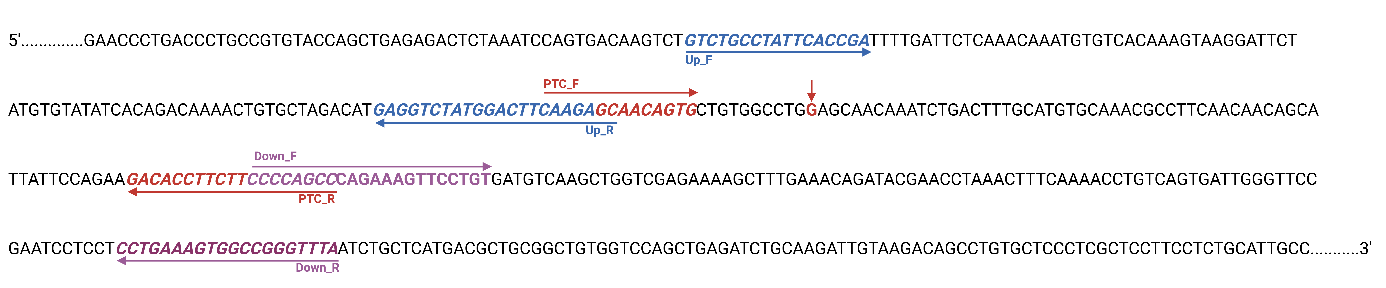


Figure S2: Primer layout relative to the PTC in the *TRAC* gene (schematic).
The sequence represents a segment of the TRAC (+) strand (GRCh38); not to scale. The red caret marks the pathogenic variant (c.194G>A; p.Trp65*; premature termination codon, PTC). Three amplicon sets with 5′→3′ orientation was shown: UP (blue; upstream of the variant), PTC (red/purple; spanning the PTC), and Down (pink/magenta; downstream of the variant. Primer names (Up_F/Up_R, PTC_F/PTC_R, Down_F/Down_R) were indicated next to the arrows.


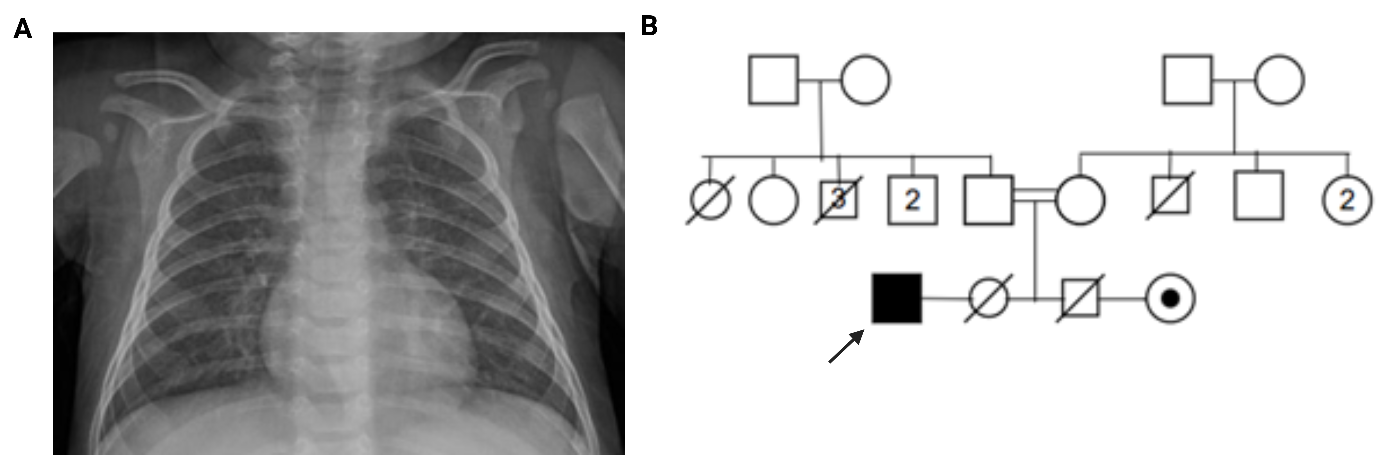


Figure S3. Direct chest X-Ray and pedigree of the patient. (A) Chest X-Ray of the index case showing absence of thymus. (B) Pedigree of the patients.


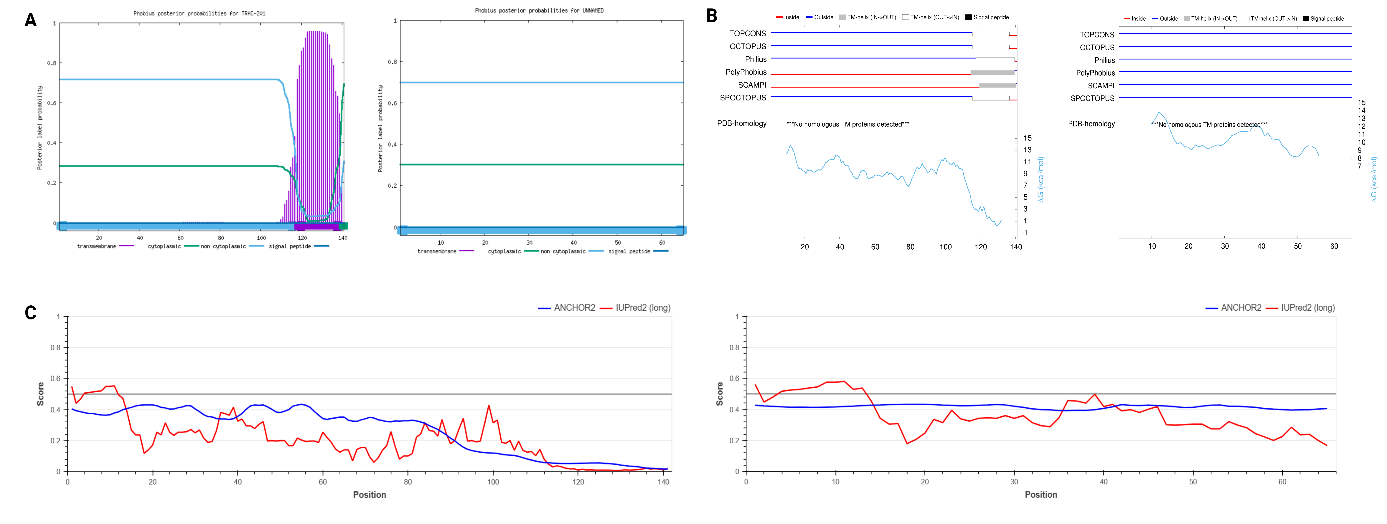


Figure S4. In-silico topology and disorder predictions for the TRAC p.Trp65* variant. (A) Phobius posterior probability plots. The wild-type TRAC sequence (left) shows a predicted non-cytoplasmic domain (aa 1–117), a transmembrane helix (aa 118–139, purple), and a short cytoplasmic tail (aa 140–141). In contrast, the truncated sequence (right; aa 1–64) lacks any predicted transmembrane region, consistent with loss of membrane anchoring. (B) TOPCONS consensus topology predictions. The wild-type sequence is predicted to contain a single transmembrane helix by multiple sub-predictors, while the truncated sequence is consistently classified as non-transmembrane. No signal peptide was predicted in either case. (C) IUPred2A/ANCHOR2 disorder plots. Wild-type TRAC shows a largely ordered profile with low disorder over the transmembrane helix and no strong MoRF signals. The truncated sequence shows only modest N-terminal disorder but lacks the ordered transmembrane segment present in WT.
